# OB-001 Enhances Osimertinib Brain Penetration: Preclinical Pharmacokinetics and Translational Rationale for EGFR-Mutant NSCLC with CNS Disease

**DOI:** 10.1158/2767-9764.CRC-25-0553

**Published:** 2026-05-26

**Authors:** Hitesh B. Mistry, Jim Millen, Josh Fleet, Mark Brimble

**Affiliations:** OncoBayesAlpha Ltd., London, United Kingdom.

## Abstract

Osimertinib is a third-generation epidermal growth factor receptor (EGFR) tyrosine kinase inhibitor with proven efficacy in EGFR-mutant non–small cell lung cancer (NSCLC), including patients with central nervous system (CNS) disease. However, pharmacokinetic (PK) studies demonstrate low CNS penetration (CSF:plasma 0.8%–22%), largely due to ABCB1/ABCG2 efflux. OB-001, a KinetiSol amorphous solid dispersion formulation of elacridar, was developed to overcome the poor bioavailability of crystalline elacridar and evaluated as a strategy to enhance osimertinib brain delivery. In CD-1 mice, we compared the oral bioavailability of OB-001 (10, 30, 100 mg/kg) with crystalline elacridar (100 mg/kg) and assessed osimertinib (50 mg/kg orally) brain distribution after pretreatment with OB-001 (10 or 50 mg/kg) or crystalline elacridar (100 mg/kg). Plasma and perfused brain PK parameters were derived by noncompartmental analysis. AUC_last_ values were compared using Bailer randomization test. OB-001 achieved greater systemic elacridar exposure than the crystalline methylcellulose suspension (*P* < 0.001). In vehicle-pretreated mice, osimertinib produced a brain:plasma AUC ratio of 7.1. OB-001 pretreatment increased brain AUC_last_ 4-fold (10 mg/kg; *P* < 0.001) and 9.3-fold (50 mg/kg; *P* < 0.001) with minimal plasma change (1.1–1.4-fold). Crystalline elacridar at 100 mg/kg increased brain AUC_last_ 5-fold (*P* < 0.001). Thus, OB-001 at 10 mg/kg achieved comparable brain enhancement with 10-fold higher dose of crystalline elacridar, whereas OB-001 at 50 mg/kg exceeded it. Translational benchmarking against *in vitro* potency suggested that raising estimated Kp,uu from ∼0.15 to ∼0.60 could improve efficacy. OB-001 selectively boosts osimertinib brain exposure while sparing systemic PK. These preclinical data support further evaluation of OB-001 as a strategy to enhance the CNS efficacy of osimertinib in EGFR-mutant NSCLC.

**Significance:**

Blood–brain barrier (BBB) efflux limits osimertinib brain exposure. Oral OB-001 pretreatment markedly increases brain concentrations with minimal plasma impact, supporting BBB transporter inhibition as a strategy to enhance CNS drug delivery.

## Introduction

Osimertinib is a third-generation, irreversible epidermal growth factor receptor (EGFR) tyrosine kinase inhibitor (TKI) that selectively targets activating mutations (exon 19 deletions, L858R) and the resistance mutation T790M, while sparing wild-type (WT) EGFR (1, 2). In the phase III FLAURA trial, osimertinib improved progression-free survival (PFS) compared with first-generation EGFR TKIs, with overall survival (OS) extended to ∼38 to 40 months (3). Intracranial efficacy was evident, with a central nervous system (CNS) objective response rate (ORR) of ∼66% and a hazard ratio for CNS progression of 0.48 (4). In the AURA3 trial, osimertinib achieved a CNS ORR of ∼70% among patients with baseline brain metastases (5). In BLOOM, osimertinib at 160 mg showed meaningful activity in leptomeningeal disease with a median intracranial PFS of 8.6 months (6). Most recently, the BLOSSOM study reported a median OS of 15.6 months in leptomeningeal patients on osimertinib 80 mg and a cerebrospinal fluid (CSF):free plasma ratio of ∼22% (7).

Despite these advances, clinical pharmacokinetic (PK) data demonstrate low and heterogeneous CNS penetration. BLOOM reported ∼16% CSF:plasma penetration (6), BLOSSOM confirmed ∼22% (7), and the OCEAN study in radiotherapy-naïve CNS metastases reported a plasma:CSF penetration of ∼0.8% (8). PET studies with [^11^C]osimertinib in healthy volunteers (9) and in EGFR-mutant patients with non–small cell lung cancer (NSCLC; ref. 10) confirmed rapid but quantitatively low brain distribution, consistent with a brain-to-plasma unbound partition coefficient (Kp,uu) well below unity.

Mechanistic studies show that osimertinib and its metabolite AZ5104 are substrates of efflux transporters ABCB1 (P-gp) and ABCG2 (BCRP). In double knockout mice, osimertinib brain exposure increased ∼6-fold compared with WT, implicating transporter efflux as a principal barrier (11).

OB-001 is a proprietary amorphous solid dispersion (ASD) of elacridar produced using KinetiSol processing, a solvent-free, high-shear technology designed to improve solubility and oral bioavailability. KinetiSol has demonstrated marked improvements in dissolution and systemic exposure for poorly soluble agents, including vemurafenib, ritonavir, and itraconazole (12–15). An earlier study demonstrated that an amorphous spray-dried dispersion of elacridar could improve oral bioavailability in human volunteers, providing precedent for the formulation-based approach (16).

In this study, we evaluated whether OB-001 could enhance osimertinib brain distribution in mice. Three complementary studies were conducted: (i) a comparison of the relative oral bioavailability of OB-001 versus crystalline elacridar (study 1), (ii) assessment of osimertinib brain penetration with OB-001 pretreatment at two dose levels (study 2a), and (iii) a direct comparison of osimertinib brain distribution following pretreatment with either OB-001 or crystalline elacridar (study 2b).

## Materials and Methods

### Study 1 – relative bioavailability of OB-001 versus crystalline elacridar

#### Animals and husbandry

Male CD-1 mice [8–12 weeks old, 32–37 g; Hylasco (RRID: IMSR_CRL:022)] were housed under standard conditions (22°C ± 3°C, 30%–70% relative humidity, and 12:12 hours light–dark cycle) with *ad libitum* access to food and water. Mice were fasted for ∼5 to 6 hours prior to dosing, with food returned 4 hours after.

#### Dosing and formulations

OB-001 was dosed orally at 10, 30, or 100 mg/kg in 0.5% (w/v) methylcellulose K4M, pH 4.0 (1 or 5 mg/mL suspensions). Crystalline elacridar was dosed orally at 100 mg/kg in 0.5% (w/v) methylcellulose, pH 4.0 (10 mg/mL). All formulations were prepared fresh, administered at 10 mL/kg, and stirred continuously during dosing. This simple methylcellulose vehicle was chosen deliberately to avoid excipients (e.g., Tween-80, Cremophor EL) that may themselves modulate P-gp or BCRP activity, which could confound interpretation of transporter inhibition studies.

#### Sampling

Blood was collected at 0.25, 0.5, 1, 2, 4, 8, and 24 hours after dose (*n* = 3/time point). Plasma was separated and stored at −70°C until analysis.

#### Bioanalysis

Plasma elacridar concentrations were determined by a fit-for-purpose LC–MS/MS method using verapamil as internal standard. Chromatography was performed on a Kinetex C18 column (100 × 3 mm, 5 μm), with mobile phases of 5 mmol/L ammonium acetate (aqueous) and 0.1% formic acid in acetonitrile at 0.8 mL/minute. Detection used positive-ion electrospray ionization in MRM mode with transitions m/z 564.20→252.10 (elacridar) and 455.10→165.10 (verapamil). Calibration was linear (2–10,000 ng/mL; 1/x^2^ weighting; r^2^ > 0.98).

#### PK analysis

Noncompartmental analysis [NCA; Phoenix WinNonlin 8.2 (RRID: SCR_024504)] was used to derive C_max_, T_max_, and AUC_last_. Dose-normalized AUC (AUC/dose) was calculated for relative bioavailability. Statistical comparisons of AUC_last_ between groups were made using Bailer randomization test for serial-sacrifice designs (17).

### Study 2 – osimertinib brain distribution with elacridar pretreatment

#### Animals and husbandry

Conditions were as in study 1.

#### Dosing and formulations

In study 2a (SYN/DMPK/2025/M0287), mice received oral OB-001 at 10 or 50 mg/kg or vehicle (0.5% methylcellulose K4M, pH 4.0) 2 hours prior to osimertinib. In study 2b (SYN/DMPK/2025/M0444), mice received oral crystalline elacridar at 100 mg/kg (in 0.5% methylcellulose, pH 4.0) 2 hours prior to osimertinib. In all groups, osimertinib was dosed orally at 50 mg/kg in 1% Tween-80 + 0.5% HPMC suspension (10 mg/mL, 5 mL/kg).

#### Sampling

At 0.25, 0.5, 1, 2, 4, 8, and 24 hours after osimertinib, mice (*n* = 3/timepoint) were anaesthetized. Blood was collected by cardiac puncture into K_2_-EDTA tubes. Following transcardial perfusion with saline, brains were excised, weighed, snap-frozen, and homogenized in phosphate buffer (2× tissue weight; final homogenate 3× tissue volume).

#### Bioanalysis

Plasma and brain concentrations of osimertinib were determined by LC–MS/MS as described in study 1. Calibration curves (2–10,000 ng/mL; r^2^ > 0.98) were accepted if ≥75% of standards (including lower limit of quantification and upper limit of quantification) were within ±20%.

#### PK analysis

NCA (Phoenix WinNonlin 8.2) derived C_max_, T_max_, and AUC_last_. Brain:plasma ratios (B/P) were calculated for both C_max_ and AUC_last_. Fold changes versus vehicle-pretreated mice were determined. Statistical comparisons of AUC_last_ used Bailer randomization test.

### Ethics approval

Both studies were performed at Syngene International Ltd., Bangalore, under Institutional Animal Ethics Committee approvals (SYNGENE/IAEC/1632/10-2024 and SYNGENE/IAEC/1681/03-2025).

## Results

### Study 1 – relative bioavailability

**Figure 1.**
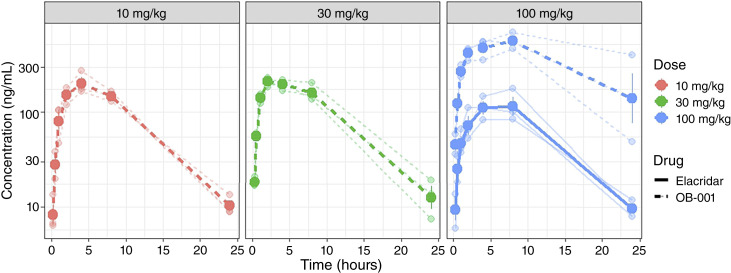
Plasma PK of OB-001 vs. crystalline elacridar after single oral dosing in CD-1 mice. Individual mouse plasma concentration–time profiles are shown as points with thin connecting lines. Group mean profiles are overlaid as thick lines, plotted on a log_10_*y*-axis. Panels are facetted by nominal dose. Line type distinguishes drug (OB-001 vs. elacridar) within each dose panel. Mice (*n* = 3 per time point per group) were sampled at 0.25, 0.5, 1, 2, 4, 8, and 24 hours after dose.

OB-001 produced substantially higher systemic elacridar exposure than the crystalline methylcellulose suspension at the same dose (Fig. 1). Following oral administration, mean plasma AUC_last_ values for OB-001 were 2,490 h·ng/mL at 10 mg/kg, 2,785 h·ng/mL at 30 mg/kg, and 9,583 h·ng/mL at 100 mg/kg. Crystalline elacridar at 100 mg/kg yielded a mean AUC_last_ of only 1,797 h·ng/mL. Thus, OB-001 at just one-tenth the dose of crystalline elacridar achieved 1.4-fold higher systemic exposure. Bailer test confirmed significantly greater AUC_last_ for OB-001 at all three doses compared with crystalline elacridar at 100 mg/kg (*P* < 0.001 for each comparison).

A notable feature of the OB-001 dose–exposure relationship was its nonlinearity. The 3-fold dose increase from 10 to 30 mg/kg produced only a 1.12-fold increase in AUC_last_, whereas the subsequent 3.3-fold increase from 30 to 100 mg/kg yielded a 3.44-fold increase. This pattern may reflect saturation of intestinal ABCB1-mediated efflux at higher local elacridar concentrations—as elacridar is itself a P-gp substrate—or dose-dependent supersaturation behavior of the ASD formulation.

It is important to note that the crystalline elacridar plasma concentrations observed in this study (mean C_max_ ∼125 ng/mL at 100 mg/kg) were lower than those reported in some published mouse studies using different formulations. Ward and Azzarano (18) reported C_max_ values approaching 600 ng/mL at 30 mg/kg, and Sane and colleagues (19) and Kemper and colleagues (20) reported similarly higher exposures. This discrepancy likely reflects the suboptimal dissolution properties of our simple methylcellulose suspension compared with the vehicles used in those studies and possibly differences in crystalline particle size. The OB-001 plasma levels, by contrast, were broadly consistent with published elacridar exposure in mice.

### Study 2 – osimertinib brain distribution with OB-001

**Figure 2.**
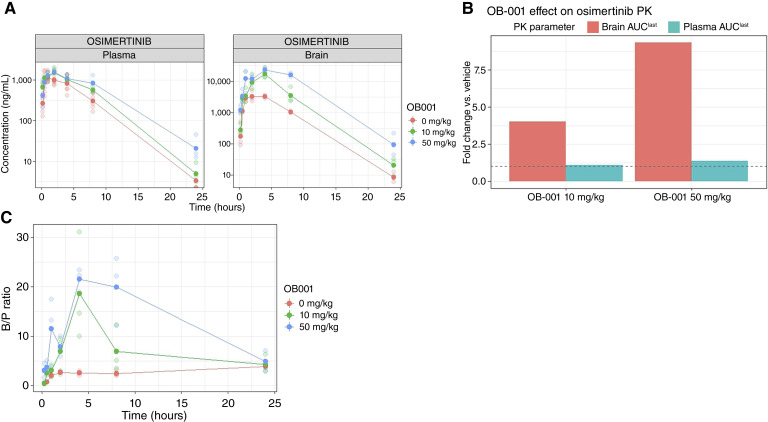
Plasma and brain PK of osimertinib with OB-001 pretreatment in CD-1 mice. **A,** Individual mouse concentration–time data are shown as points, with group means overlaid as lines on a log_10_*y*-axis. Panels separate plasma and perfused brain concentrations. Colors denote OB-001 pretreatment dose: 0 mg/kg (vehicle), 10 mg/kg, or 50 mg/kg, administered orally 2 hours prior to osimertinib (50 mg/kg orally). Mice (*n* = 3 per time point per group) were sampled at 0.25, 0.5, 1, 2, 4, 8, and 24 hours after osimertinib. **B,** Fold change in osimertinib plasma and brain AUC_last_. Bars show changes in plasma AUC_last_ and brain AUC_last_ at 10 and 50 mg/kg OB-001, normalized to the vehicle (0 mg/kg) control. The dashed line indicates no change (fold change = 1). **C,** Individual mouse brain to plasma concentration ratio–time data are shown as points, with group means overlaid as lines. Colors denote OB-001 pretreatment dose: 0 mg/kg (vehicle), 10 mg/kg, or 50 mg/kg, administered orally 2 hours prior to osimertinib (50 mg/kg orally). Mice (*n* = 3 per time point per group) were sampled at 0.25, 0.5, 1, 2, 4, 8, and 24 hours after osimertinib.

OB-001 pretreatment markedly and selectively enhanced osimertinib brain exposure (Fig. 2). In vehicle-pretreated mice, osimertinib 50 mg/kg produced a plasma C_max_ of 1,555 ng/mL (T_max_ 0.5 hours) and AUC_last_ of 11,825 h·ng/mL. OB-001 pretreatment at both dose levels preserved osimertinib plasma exposure: AUC_last_ was 12,756 h·ng/mL with OB-001 10 mg/kg (1.08-fold) and 16,013 h·ng/mL with OB-001 50 mg/kg (1.35-fold).

**Figure 3.**
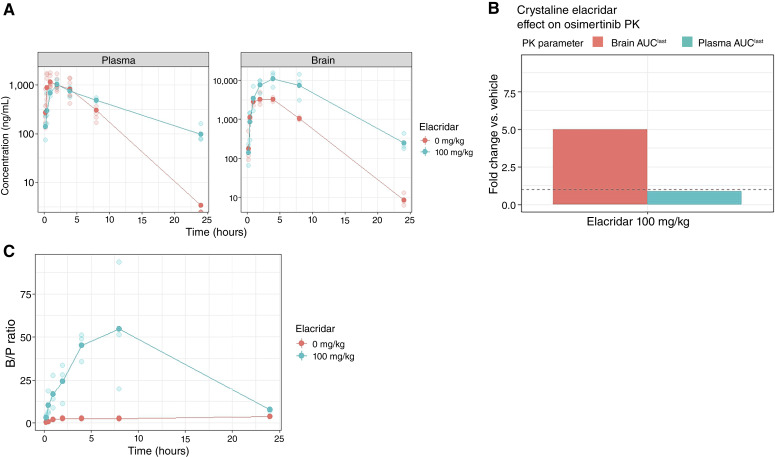
B/P ratio of osimertinib with crystalline elacridar pretreatment in CD-1 mice. **A,** Individual mouse concentration–time data are shown as points, with group means overlaid as lines on a log_10_*y*-axis. Panels separate plasma and perfused brain concentration. Colors denote 0 mg/kg (vehicle) and 100 mg/kg crystalline elacridar, administered orally 2 hours prior to osimertinib (50 mg/kg orally). Mice (*n* = 3 per time point per group) were sampled at 0.25, 0.5, 1, 2, 4, 8, and 24 hours after osimertinib. **B,** Fold change in osimertinib plasma and brain AUC_last_. Bars show changes in plasma AUC_last_ and brain AUC_last_ at 100 mg/kg crystalline elacridar, normalized to the vehicle (0 mg/kg) control. The dashed line indicates no change (fold change = 1). **C,** Individual mouse B/P concentration ratio–time data are shown as points, with group means overlaid as lines. Colors denote pretreatment dose: 0 mg/kg (vehicle) or 100 mg/kg crystalline elacridar, administered orally 2 hours prior to osimertinib (50 mg/kg orally). Mice (*n* = 3 per time point per group) were sampled at 0.25, 0.5, 1, 2, 4, 8, and 24 hours after osimertinib.

In vehicle-pretreated mice, osimertinib brain C_max_ was 10,198 ng/g (T_max_ 2 hours) with a B/P AUC_last_ ratio of 7.1. OB-001 at 10 mg/kg increased brain C_max_ to 53,804 ng/g (5.3-fold) and brain AUC_last_ to 334,839 h·ng/g (4-fold), with the B/P AUC_last_ ratio rising to 26.3. OB-001 at 50 mg/kg further increased brain C_max_ to 69,161 ng/g (6.8-fold) and brain AUC_last_ to 777,792 h·ng/g (9.3-fold), with a B/P AUC_last_ ratio of 48.6. Bailer test confirmed significant increases in brain AUC_last_ for all OB-001 pretreatment groups versus vehicle (*P* < 0.001). OB-001 at 50 mg/kg substantially exceeded crystalline elacridar (9.3-fold vs. 5-fold). Examination of B/P concentration ratios over time, see Fig. 3, revealed that at early time points (up to 4 hours), the 10 mg/kg OB-001 group achieved B/P ratios approaching those of the 50 mg/kg group. However, at later time points (8–24 hours), the 50 mg/kg group maintained higher B/P ratios, consistent with more sustained transporter inhibition from higher and more prolonged elacridar exposure.

In addition to assessing osimertinib, crystalline elacridar was also studied at a dose of 100 mg/kg (Fig. 3). At that dose, osimertinib plasma exposure was numerically lower than that of the original vehicle group (C_max_ 1,039 ng/mL; AUC_last_ 10,207 h·ng/mL; 0.86-fold). Given that this arm was evaluated in a separate experiment and that only three mice were sampled per time point, this modest difference should be interpreted cautiously. Crystalline elacridar at 100 mg/kg increased osimertinib brain C_max_ to 35,252 ng/g (3.5-fold) and osimertinib brain AUC_last_ to 420,559 h·ng/g (5-fold), with a B/P AUC_last_ ratio of 41.2. Bailer test confirmed significant increases in brain AUC_last_ for the crystalline elacridar pretreatment group. Thus, OB-001 at just 10 mg/kg produced brain enhancement comparable to crystalline elacridar at 100 mg/kg (4-fold vs. 5-fold AUC_last_ increase), representing a 10-fold dose advantage.

## Discussion

Osimertinib has redefined treatment for EGFR-mutant NSCLC, including patients with CNS disease. Clinical trials (FLAURA, AURA3, BLOOM, BLOSSOM, and OCEAN) demonstrate consistent intracranial efficacy, yet PK studies reveal low and heterogeneous CNS penetration, with CSF:plasma ratios ranging from 0.8% to 22% (6–8). PET imaging corroborates low brain distribution (9, 10). Mechanistic studies implicate ABCB1/ABCG2 efflux as the primary limitation (11).

Our data demonstrate that elacridar pretreatment—whether as OB-001 or crystalline compound—markedly enhances osimertinib brain exposure in mice while largely sparing systemic plasma PK. These PK gains are consistent with selective efflux inhibition at the blood–brain barrier (BBB). The inclusion of a direct crystalline elacridar comparator in our tissue distribution study allows several important conclusions. First, OB-001 at 10 mg/kg achieved brain enhancement comparable with a 10-fold higher dose of crystalline elacridar (4-fold vs. 5-fold brain AUC_last_ increase), confirming a meaningful dose advantage. Second, OB-001 at 50 mg/kg substantially exceeded the crystalline compound (9.3-fold vs. 5-fold). Third, whereas OB-001 preserved osimertinib plasma exposure, crystalline elacridar pretreatment was associated with a modest (∼14%) reduction in osimertinib plasma AUC_last_. Because this arm was added later and each time point comprised only three animals, this numerical difference may simply reflect interexperimental variability and should not be overinterpreted as evidence of a true PK interaction. In addition, P-gp and BCRP can be expressed by tumor cells themselves, including intracranial metastases, in which they act to lower intracellular drug concentrations. By inhibiting efflux at both the BBB and within tumor cells, OB-001 is expected to raise not only the amount of osimertinib entering the brain but also the levels retained inside cancer cells.

To contextualize these results, we benchmarked free brain concentrations against *in vitro* potency, as measured via growth inhibition 50 values (GO50). Most proliferation assays are performed in medium containing 10% fetal bovine serum. Under these conditions, the protein concentration is ∼1/10 that of plasma; for highly protein-bound drugs such as osimertinib (>95% bound), the free fraction in medium (fu,media) can be approximated at 0.1 (21). Thus, GI50_free_ ≈ 0.1 × GI50_total_. This correction is conservative: if fu,serum ≈ 0.01, then fu,media ≈ 0.09 to 0.10; if fu,serum ≈ 0.05, fu,media would be higher (∼0.3–0.5). We therefore present GI50_free assuming fu,media = 0.1, with the acknowledgment that actual values may be somewhat higher. Corrected GI50 values across EGFR mutations are shown in Table 1.

**Table 1.** *In vitro* GI50 values (total vs. free corrected, fu,media = 0.1).

Mutation	GI_50_ total (nmol/L)	GI_50_ free (nmol/L)	Key sources
L858R	1–3	0.1–0.3	(1, 2)
del19	5–10	0.5–1	(1, 2)
L858R/T790M	8–20	0.8–2	(1, 24)
L861Q	8–15	0.8–1.5	(1)
G719X	12–20	1.2–2	(1)
Exon 20 insertion	80–120	8–12	(25)

At an estimated clinical baseline Kp,uu (∼0.15; ref. 8), osimertinib achieves near-maximal effect against common sensitizing mutations (L858R and del19) but is suboptimal against less common variants (T790M, L861Q, and G719X) and exon 20 insertions. OB-001 pretreatment is predicted to increase Kp,uu to ∼0.60, resulting in higher target coverage across EGFR mutations (Table 2). This analysis is hypothesis-generating and subject to substantial uncertainty in both the fu,media correction and the extrapolation of Kp,uu from mice to humans. It should be noted that increased target coverage for ALK inhibitors has led to marked improvement in CNS PFS (22). Several limitations of this work warrant discussion. First, mice exhibit substantially higher oral elacridar bioavailability than rats, dogs, monkeys, or humans. Ward and Azzarano (18) demonstrated that whereas mice achieve elacridar C_max_ values exceeding 600 ng/mL at moderate oral doses, plasma concentrations in other species plateau at much lower levels, and Kuppens and colleagues (23) confirmed that elacridar AUC and C_max_ in humans did not increase meaningfully when the oral dose was raised from 100 to 1,000 mg. This species difference means that the mouse model, although appropriate for demonstrating the principle of enhanced brain penetration via transporter inhibition, is not the ideal species for evaluating whether the ASD formulation *per se* improves oral bioavailability relative to crystalline elacridar. Definitive assessment of formulation superiority will require studies in species with poorer oral elacridar absorption or, ultimately, in humans. Encouragingly, Sawicki and colleagues (16) demonstrated that an amorphous dispersion of elacridar could improve oral bioavailability in human volunteers, providing proof-of-concept for the formulation approach, although the pill burden was too high. Second, the crystalline elacridar plasma concentrations observed in study 1 (mean C_max_ ∼125 ng/mL at 100 mg/kg) were lower than reported in some published studies (18–20), likely reflecting the simple 0.5% methylcellulose vehicle and possibly coarser particle size. The OB-001 plasma levels, by contrast, were consistent with published elacridar concentrations in mice. Although this limits the strength of the formulation comparison in study 1, the head-to-head tissue distribution comparison in study 2b provides direct functional evidence that OB-001 achieves superior brain enhancement at substantially lower doses.

**Table 2.** Predicted %effect (Emax model, Cavg,ss ≈ 20 nmol/L free plasma; refs. 5, 7).

Mutation	Baseline Kp,uu = 0.15	With OB-001 Kp,uu = 0.60
L858R	∼90%	∼98%
del19	∼80%	∼94%
L858R/T790M	∼68%	∼90%
L861Q	∼70%	∼91%
G719X	∼65%	∼88%
Exon 20 insertion	∼20%	∼55%

Thus, OB-001 has the potential not only to broaden osimertinib’s efficacy across mutation subtypes but more importantly has the potential to translate into prolonged CNS PFS, as seen with ALK inhibitors (22). CNS progression remains a major site of failure on first-line osimertinib (3, 4), and by increasing both brain penetration and intratumoral drug retention without altering systemic PK, OB-001 could extend the durability of intracranial control beyond the ∼16 to 19 months reported in current trials. If confirmed clinically, such an approach could reduce CNS relapse rates and delay the need for local therapies such as radiotherapy, representing a meaningful advance in the management of EGFR-mutant NSCLC.

## Data Availability

The data generated in this study are available upon request to the corresponding author.
